# Association of diabetes risk with changes in memory, working memory, and processing speed among older adults

**DOI:** 10.3389/fpsyg.2024.1427139

**Published:** 2024-11-12

**Authors:** Jungjoo Lee, Junhyoung Kim, Sang Joon An

**Affiliations:** ^1^School of Health Professions, College of Nursing and Health Professions, University of Southern Mississippi, Hattiesburg, MS, United States; ^2^Department of Health Behavior, School of Public Health, Texas A&M University, College Station, TX, United States; ^3^Department of Neurology, The Convergence Institute of Healthcare and Medical Science, International St. Mary’s Hospital, Catholic Kwandong University, Incheon, Republic of Korea

**Keywords:** older adults, risk of diabetes, memory, working memory, processing speed

## Abstract

**Background:**

This study investigated the risk of diabetes by examining changes in memory, working memory, and processing speed among older adults to provide evidence on how each cognitive domain is associated with the risk of diabetes in older adults.

**Methods:**

This study used Health and Retirement Study data and tracked the respondents from 2012 to 2020 (*n* = 5,748). The Telephone Interview for Cognitive Status-27 includes three cognitive tests (recall, seven subtraction, and counting backward tests) to assess each cognitive domain. A Cox proportional hazard regression was used to calculate the changes in the odds ratio (OR) of diabetes by increasing each cognitive function and the parameter in covariates.

**Results:**

We found that the OR of diabetes decreased with increasing universal cognitive function, increasing memory, working memory, and processing speed, and that age increased the OR in all analysis models.

**Conclusion:**

The findings of this study contribute to filling gaps in the literature by exploring: (a) the association between each cognitive function and the decline in diabetes risk and (b) the varying patterns of change in diabetes risk with increasing cognitive function.

## Introduction

Diabetes is a serious chronic disease affecting the geriatric population that has been associated with aggravating comorbidity risks, including cardiovascular disease and neuropsychological and cognitive disorders (Campbell et al., 2012; Fang et al., 2019; Gregg et al., 2018; Gregg et al., 2014; Gordon-Dseagu et al., 2014; Seshasai et al., 2011). An estimated 33% of older adults have diabetes and are at higher risk of developing diabetes-related comorbidities, including mellitus control issues, kidney failure, and heart disease (Care, 2018; Saeedi et al., 2020; World Health Organization, 2023). The risk of diabetes and related diseases is projected to increase by 6.1% each year through 2035, with further increase expected as the U.S. population continues to age (World Health Organization, 2023).

A growing body of literature reports a relationship between cognitive decline and the risk of diabetes among older adults (Antal et al., 2022; Willmann et al., 2020; Zhao et al., 2017; Zheng et al., 2018). These studies have reported that diabetes has been associated with accelerated cognitive decline. Researchers have reported that insulin resistance caused by diabetes impacts both cerebrovascular and non-cerebrovascular pathways and results in cognitive decline among older adults with diabetes (Matsuzaki et al., 2010; Palta et al., 2018). Thus, it is suggested that older adults with diabetes are likely to experience significant cognitive decline, possibly leading to Alzheimer’s disease and related dementias (ADRDs) (Lu et al., 2009; Willette et al., 2015).

The relationship between diabetes and cognitive decline is well-documented. Studies have highlighted that diabetes drastically accelerates cognitive decline, particularly in the older population (Wessels et al., 2011; Ravona-Springer et al., 2010; Maggi et al., 2009). In a 15-year follow-up study of elderly African Americans, diabetes was found to hasten cognitive deterioration (Wessels et al., 2011). In contrast, another study showed that older adults with dementia and diabetes experienced faster cognitive decline than those without diabetes (Ravona-Springer et al., 2010). Additionally, diabetes was identified as a risk factor for cognitive decline in older patients, with higher HbA1c levels being associated with poorer memory performance (Maggi et al., 2009).

While the results of several meta-analyses have indicated that older adults with diabetes exhibit reduced processing speed, executive functioning, and motor control than those without diabetes (Wessels et al., 2011; Ravona-Springer et al., 2010; Maggi et al., 2009; Debette et al., 2011), there is, currently, a dearth of evidence describing which cognitive domains (e.g., memory, word processing, attention, and executive function are affected by diabetes) among older adults with diabetes (Spauwen et al., 2013). A longitudinal cohort study (i.e., Whitehall II) reported that diabetes was negatively associated with memory function and reasoning performance, but not related to processing and attention speed (Tuligenga et al., 2014).

Other meta-analyses have explored a reverse relationship between cognitive decline and the risk of diabetes (Spauwen et al., 2013; Tuligenga et al., 2014; Bangen et al., 2018; Mõttus et al., 2013). These studies focused on how changes in cognitive function are associated with the risk of developing diabetes and suggested that cognitive declines predicted the risk of diabetes among older adults, though the results of these studies have been inconsistent in their evaluation of the relationship between certain cognitive domains and the risk of diabetes in older adults. Previous studies focused only on overall cognitive function rather than specific domains such as memory, processing speed, and executive function and therefore did not provide conclusive evidence of how the cognitive domain may impact the risk of diabetes differently in older adults (Wessels et al., 2011; Ravona-Springer et al., 2010; Maggi et al., 2009; Debette et al., 2011).

Our study focused on understanding the relationship between different cognitive function domains and the risk of diabetes. Based on previous studies (Jester et al., 2023; Tibiriçá et al., 2023; Williams et al., 2020), this study included three main cognitive domains: memory, working memory, and processing speed. Thus, the purpose of this study was to investigate the risk of diabetes by examining changes in the memory, working memory, and processing speed of older adults to provide evidence on how each cognitive domain is associated with the risk of diabetes.

## Methods

### Data source

We used the core data from the Health and Retirement Study (HRS) covering the years 2012 to 2020, which has been published biannually since 1992. The data includes a broad range of information about biofeedback, mental health, cognition, pension, and socioeconomically related characteristics of individuals over 45. The HRS core data comprises over 30 domains, each containing multiple psychological measurement scales and objective measures. The HRS uses both patient response outcome (PRO) and proxy measured outcome (PMO) methods for individuals who lack the ability to respond without support from caregivers. The study interviewer decides whether to use the PRO or PMO methods based on the interviewee’s ability to respond effectively. If the person answers reliably, the PRO method is chosen. If they are unresponsive or unreliable, the interviewer turns to the PMO method, gathering information from caregivers. Consequently, PRO data indicate that respondents are capable of providing their answers, and users can generally trust the reliability of those responses.

The HRS consists of a respondent pool and continues to track respondents from the time they enter the pool. This tracking is managed through a consented protocol that relies on a person identification number (PN) and household (HH) number. Each respondent is designated a specific PN, and the HH encompasses the family structure of the PNs. Changes in family structure are recorded for each PN. For example, if PN1 and PN2 in HH-1 experience a divorce, they are redesignated as PN1 in HH-1 and PN2 in HH-2. Similarly, if PN1 in HH-1 enters a second marriage, HH-1 would include PN1 and the other PN, who becomes the new partner.

### Study sample

**Figure 1:**
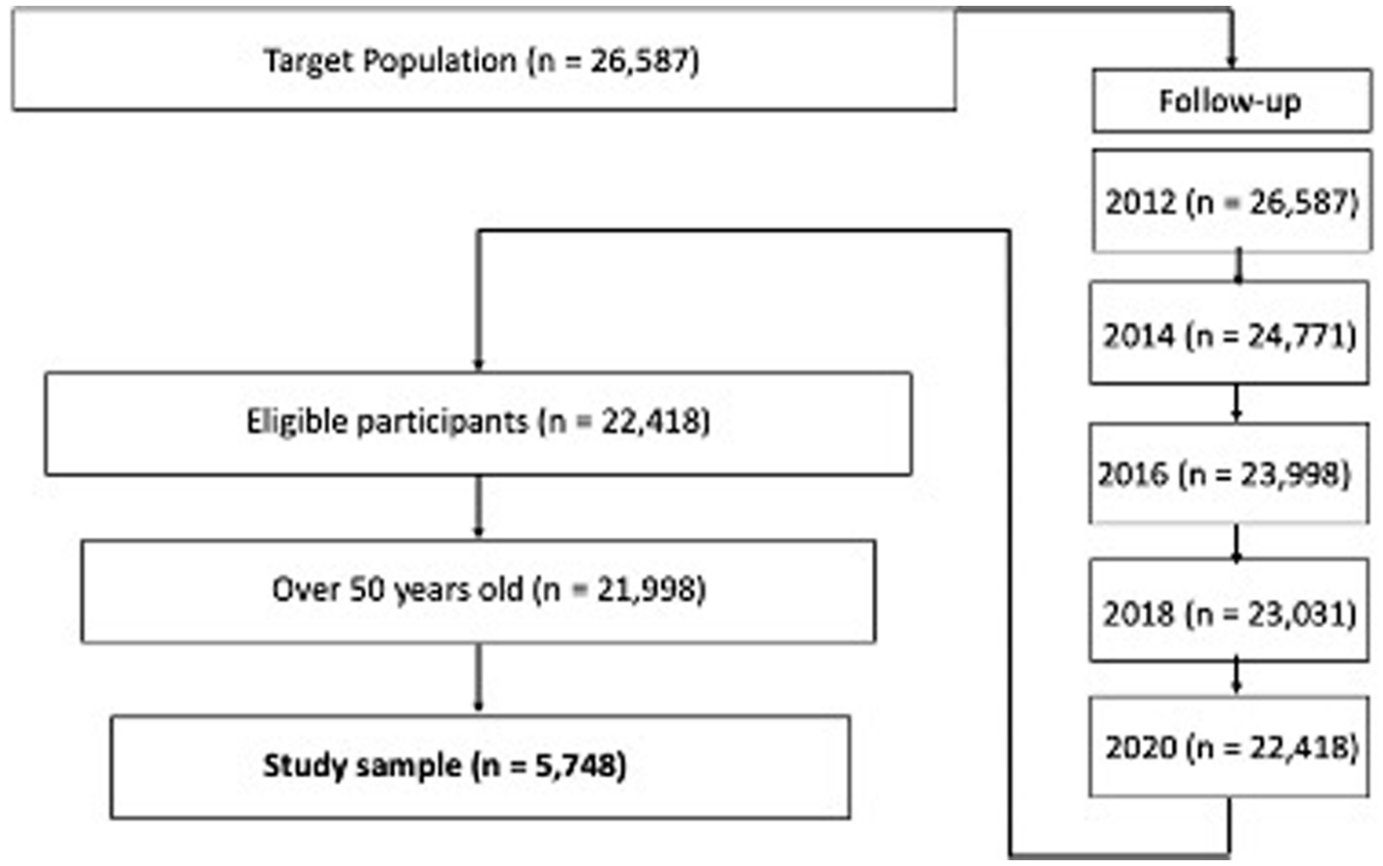
Flow chart of the longitudinal follow-up of study participants.

The baseline of the study sample extraction from the HRS respondent pool consists of the respondents included in the 2012 core data (Figure 1). This study tracked the respondents from 2012 to 2020 and merged the datasets. The study sample included censored data because of factors such as death, medical records, and personal information. While the missing value treatment used has been controversial, this study excluded missing study variable values rather than using multivariate imputation by chained equation (MICE). Even though the HRS provided information about the study samples, it did not provide information about why respondents dropped out and/or did not respond. The arbitrary imputation of a calculated value on a missing value not only reduces the power of the model explanation but also causes unexpected bias due to a lack of information about the missing values (Kang, 2013; Song and Shepperd, 2007). Thus, the total study sample for this research was 5,748.

### Instruments

#### Dependent variable

##### Diabetes

Diabetes onset was assessed by a question item: “Has a doctor told you that you have diabetes?” to which respondents answered either “Yes” or “No.” Each answer was coded as 1 = ‘Yes’ and 0 = ‘No’. The data did not include information about whether the diagnosed diabetes was type 1 or type 2. The HRS provides further information about the time of diabetes onset by an item: “In what year was your diabetes first diagnosed?” This study only included respondents who were diagnosed between 2012 and 2020.

#### Independent variable

##### Cognitive function

The cognition section in the HRS encompassed both PRO and PMO data. The HRS interviewer chose the assessment type at the time of the interview depending on whether the respondent was able to complete the testing without support from a caregiver. For this study, we used only the PRO data, which indicate that the respondents were able to complete the testing without caregiver support. Both PRO and PMO assessments use the Telephone Interview for Cognitive Status-27 (TICS-27), which comprises three types of cognitive tests: recall exercises, seven subtraction problems, and backward counting tasks. The TICS-27 has been recognized to be both valid and reliable for assessing cognitive status in large cohort studies (Crimmins et al., 2016; Herzog and Wallace, 1997; Crimmins et al., 2010).

Among a variety of cognition tests in the section, we used three cognitive function tests to assess memory, working memory, and attention and processing speed (Crimmins et al., 2016; Herzog and Wallace, 1997; Crimmins et al., 2010). First, the recall test was used to assess memory and delayed memory functions. The HRS interviewer showed respondents 10 random words and asked them to recall them immediately and 5 min later (e.g., tree, cloud, and sky). The respondents gained one point for each correct word, resulting in a total memory function score ranging from 0 to 20. The seven subtraction test was used to measure the working memory domain. The respondents were asked to subtract 7 from 100 five times (e.g., 100–7 = 93, 93–7 = 86, 86–7 = 73), earning one point for each correct answer. This resulted in a score ranging from 0 to 5, with a higher score indicating a higher level of working memory function. The counting backward test was used to measure attention and processing speed (Fisher et al., 2013; Ofstedal et al., 2005). Respondents were required to count backward from 20 to 10 continuously twice (e.g., 20, 19, 18, 17). Each correct trial had a value of one point, resulting in a total score ranging from 0 to 2. The total scores of the three cognitive function tests ranged from 0 to 27, with a higher score indicating a higher level of cognitive function.

#### Covariates

##### Age and sex

To achieve valid and reliable estimates of the coefficients of the predictors, it is crucial to leverage covariates that possess two key characteristics: they should be predictable with regard to the study outcomes and exhibit minimal correlation with the variable whose coefficient is being estimated. This approach improves the precision of coefficient estimation (Strawbridge and Wallhagen, 1999). Several covariates have been considered in previous studies on diabetes, including age, sex, weight, and height (Vagelatos and Eslick, 2013; van Manen et al., 2007; Zhang et al., 2022). As age and sex data were available in the HRS dataset, we chose to incorporate them as covariates in our study. These specific covariates were selected due to their availability in the dataset and their fulfillment of the aforementioned criteria. By including age and sex as covariates, we accounted for their potential influence on the study outcomes, thereby minimizing any confounding effects they might introduce. Ultimately, this approach contributes to a more robust and accurate estimation of the coefficient of the predictor variable under investigation.

##### Analysis

The primary purpose of this study was to investigate the relationship between the odds ratio (OR) of diabetes, cognitive functions, and covariates. Using a Cox proportional hazards regression, we assessed how increases in each cognitive function and various covariate parameters affect the risk of diabetes. The results of the respective cognitive functions were visualized. All statistical analyses were conducted using the SPSS 29.0 statistical package. The regression equation we used is provided below:

$$\begin{array}{l} \mathrm{Odds ratio}\;\left(\mathrm{OR}\right)=\frac{\mathrm{Onset of diabetes}}{1-\mathrm{Onset of diabetes}}\\ =\mathrm{Constant}+\mathrm{Age}+\mathrm{Sex}+\mathrm{Cognitive Function} \end{array}$$

$$\begin{array}{l} \mathrm{OR of diabetes}=\beta_{0}+\{\left(\mathrm{Age}\;\beta_{1}+\mathrm{Sex}\;\beta_{2}\;\right)+(\mathrm{Memory}\;\beta_{3}\\ \;\mathrm{+Working Memory}\;\beta_{4}+\mathrm{Processing Speed}\;\beta_{5})\} \end{array}$$

## Results

Table 1 describes the demographic information of respondents. The age of the study population ranged from 50 to 109 (*M* = 76.19, SD = 11.83), with 40.3% (*n* = 2,316) of the study population identifying as male and 59.6% as female (*n* = 3,432). Over half of the respondents reported their marital status as married (61.0%, *n* = 3,506), with the bulk of the remaining respondents reporting being widowed (31.1%, *n* = 1,790). The educational levels were as follows: 43.9% (*n* = 1,563) had a bachelor’s degree, 27.2% (*n* = 2,523) had less than a bachelor’s degree, 11.2% (*n* = 643) held a master’s degree, 3.1% (*n* = 178) fell into “Others,” and 14.6% (*n* = 841) had unknown education. The descriptive statistics of study variables are presented in Table 2. Of the 5,748 respondents, 769 were reported to be diagnosed with diabetes between 2012 and 2020 (13.4%, *M* = 0.13, SD = 0.34). The summed cognitive function score from 2012 to 2020 had a 67.9 mean score out of 135 (SD = 15.53). The mean score of the memory function was 48.96 out of 100 (SD = 11.4), working memory was 13.9 out of 25 (SD = 0.72), and attention and processing speed was 4.64 out of 10 (SD = 4.01).

**Table 1:** Demographic variables.

Characteristics	*n*	%
Age		
50 to 109 years old (Mean = 76.19, SD = 11.83)	5,748	100
Sex		
Male	2,316	40.3
Female	3,432	59.7
Marital status		
Married	3,506	61.0
Living with a partner	55	0.95
Divorced	65	1.1
Widowed	1790	31.1
Never married	332	5.8
Education		
Less than bachelors	1,563	27.2
Bachelors	2,523	43.9
Masters	643	11.2
Others	178	3.1
Unknown	841	14.6

Total *n* = 5,748.

**Table 2:** Descriptive statistics.

Variables	n	Mean	SD
Dependent variables		0.13	0.34
Diabetes	769 (13.4%)		
Non-diabetes	4,979 (86.60%)		
Independent variables (from 2012 to 2020)			
Cognitive function		67.9	15.53
Memory		48.96	11.4
Working memory		13.9	6.73
Processing speed		4.64	0.72

Total *n* = 5,748.

Table 3 shows the results of the model coefficient tests between the OR of diabetes and respective cognitive functions. The results of the universal cognitive function model (chi-square = 10.88, df = 2, *p* < 0.05), memory model (chi-square = 2.03, df = 2, *p* < 0.05), working memory model (chi-square = 8.07, df = 2, *p* < 0.05), and processing speed model (chi-square = 7.56, df = 2, *p* < 0.05) were all significant, indicating that the OR of diabetes declined with increasing cognitive functions.

**Table 3:** Model coefficients tests.

	Overall			Change from Previous Block		
−2 Log likelihood	Chi-square	df	Sig.	Chi-square	df	Sig.
Universal cognition						
11689.91	10.88	2	0.00*	10.67	2	0.00*
Memory						
12022.45	2.03	2	0.00*	19.56	2	0.00*
Working memory						
16892.96	8.07	2	0.02*	7.99	2	0.01*
Processing speed						
17877.05	7.56	2	0.02*	7.51	2	0.02*

^*^p < 0.05.

**Figure 2:**
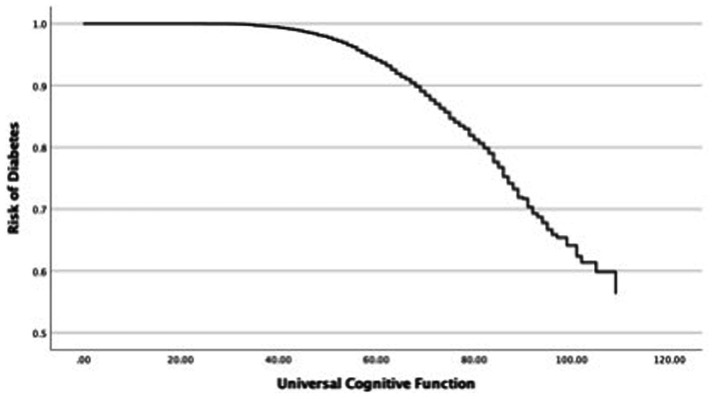
Risk changes in diabetes: universal cognitive function.

**Figure 3:**
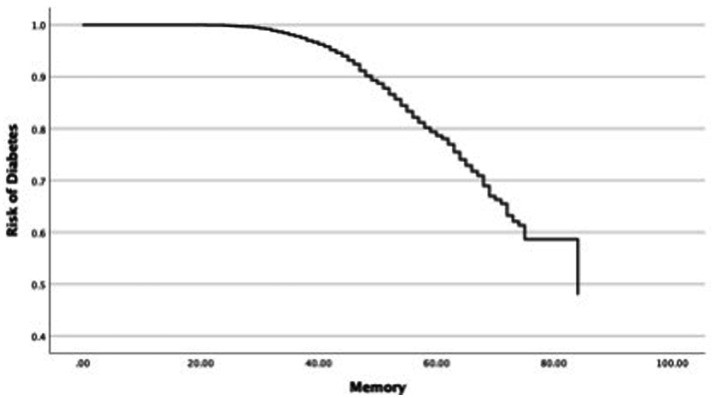
Risk changes in diabetes memory.

**Figure 4:**
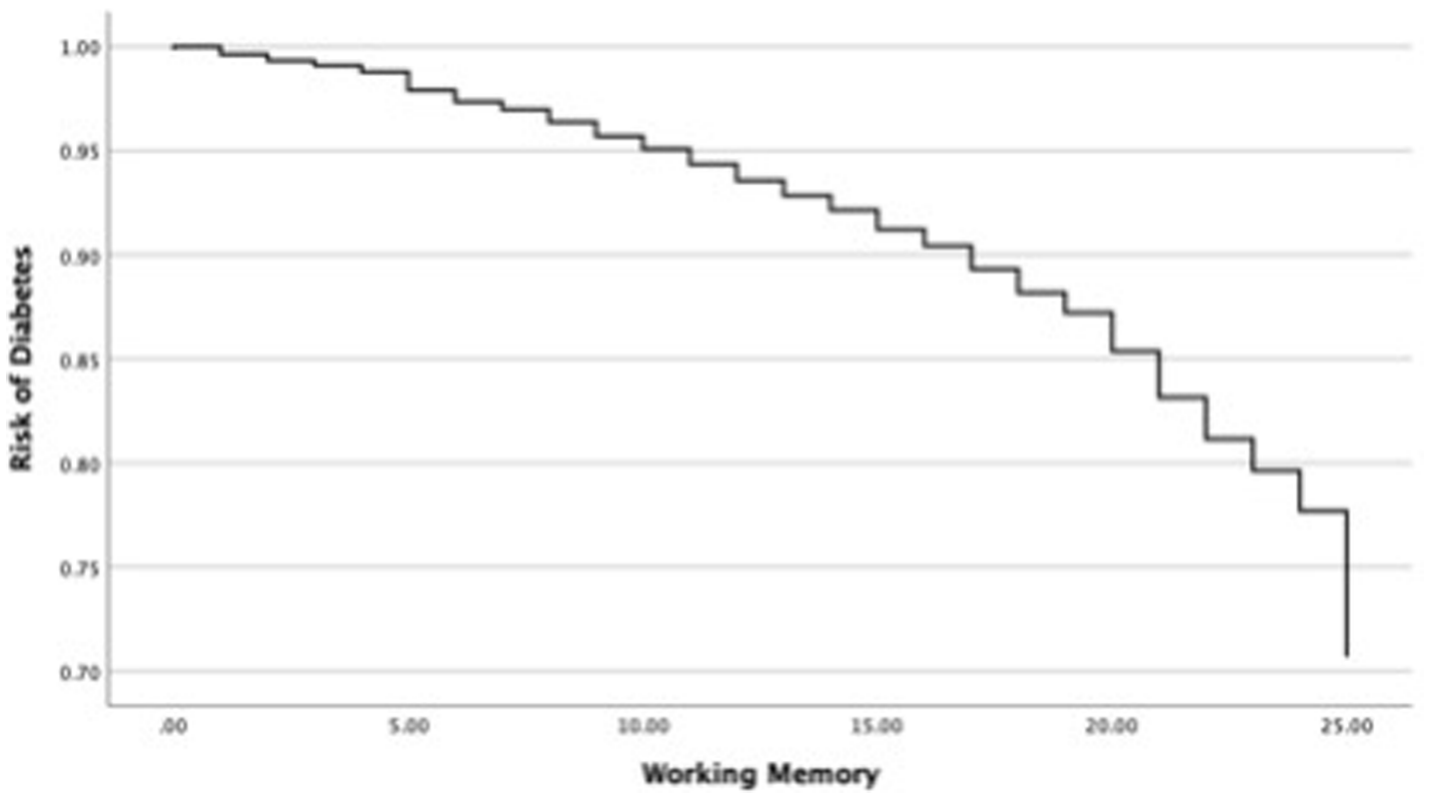
Risk changes in working memory.

**Figure 5:**
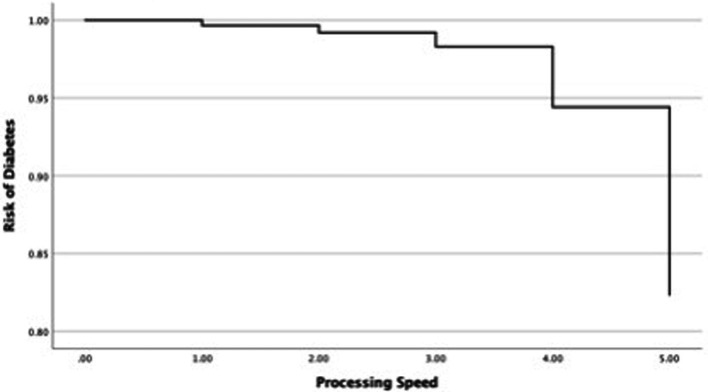
Risk changes in diabetes: processing speed.

In the next step, this study investigated changes in the OR of diabetes in each cognitive function and covariates such as age and sex (Table 4). First, the relationship between the OR of diabetes and universal cognitive function showed that the OR decreases with increasing universal cognitive function (Figure 2). The slope coefficient began to increase at a universal cognitive score of 40, and the OR of diabetes linearly decreased between 40 and 100 of the universal cognitive score. However, the OR of diabetes did not decrease until the universal cognitive function reached a score of 40. Age (OR = 1.01, Wald = 10.84, *p* < 0.05, 95% CI: 1.00–1.02) was a significant estimator of an increased OR in the aging progress. Second, the OR of diabetes decreased with increasing working memory (Figure 3). The slope coefficient commenced its ascent at a memory score of 35 and remained consistently linear until reaching a score of 80. However, the OR of diabetes did not decline until the memory function attained a score of 30. In the declining slope, age (OR = 1.01, Wald = 19.75, *p* < 0.05, 95% CI: 1.00–1.02) was as significant in increasing the OR of diabetes as was aging. Third, the OR of diabetes presented a declining slope with increasing working memory (Figure 4). The declining slope of the diabetes risk exhibited a stepwise pattern with increasing working memory. Age (OR = 1.01, Wald = 7.95, *p* < 0.05, 95% CI: 1.00–1.01) was significant in increasing the OR of diabetes. Fourth, the OR of diabetes was found to have a declining slope with increasing processing speed (Figure 5). The OR of diabetes exhibited a decreasing slope, with the maximum decrease amounting to 6%, even when the processing speed reached its highest score of 5. We found that age (OR = 1.01, Wald = 6.70, *p* < 0.05, 95% CI: 1.00–102) was a significant estimator in raising the OR of diabetes. In summary, the OR of diabetes decreased with increasing overall cognitive function, memory, working memory, and processing speed, while age was associated with an increase in the OR as cognitive function declined.

**Table 4:** Risk of diabetes and cognitive functions.

	B	SE	Wald	df	Sig.	Exp(B)	95% CI for Exp (B)	
							Lower	Upper
Universal cognition								
Age	0.01	0.00	10.84	1	0.00*	1.01	1.00	1.02
Sex	0.01	0.07	0.03	1	0.86	1.02	0.87	1.17
Memory								
Age	0.01	0.00	19.75	1	0.00*	1.01	1.00	1.02
Sex	−0.03	0.07	0.22	1	0.64	0.96	0.84	1.12
Working memory								
Age	0.01	0.00	7.95	1	0.00*	1.01	1.00	1.01
Sex	−0.02	0.06	0.16	1	0.69	0.98	0.87	1.11
Processing speed								
Age	0.01	0.00	6.70	1	0.01*	1.01	1.00	1.01
Sex	−0.06	0.06	0.87	1	0.35	0.94	0.94	1.07

^*^p < 0.05.

## Discussion

We investigated changes in the risk of diabetes associated with memory, working memory, and processing speed among older adults. The overall findings of this study are that an increase in cognitive function was associated with a decrease in the risk of diabetes, with varying slopes in the risk reduction observed in each cognitive domain (memory, working memory, and processing speed). This finding highlights the impact of cognitive function on the risk of diabetes and provides much-needed evidence for studies involving older adults and diabetes.

The majority of previous studies investigated the impact of diabetes on cognitive decline and cognitive impairment among older adults (Campbell et al., 2012; Fang et al., 2019; Gregg et al., 2018; Gregg et al., 2014; Gordon-Dseagu et al., 2014; Seshasai et al., 2011). Other studies demonstrated the effect of universal cognitive function on the risk of diabetes and provided inconsistent findings on this relationship (Spauwen et al., 2013; Tuligenga et al., 2014; Xue et al., 2019). For example, Tuligenga et al. (2014) who conducted longitudinal research, reported that diabetes was associated with decreasing memory and reasoning function but not related to processing and attention speed. On the other hand, a 12-year longitudinal study conducted by Spauwen et al. (2013) demonstrated that diabetes is associated with memory function loss as well as significant impairments in attention and processing speed. Our study presented evidence for a longitudinal relationship between two cognitive aspects (overall cognitive function and individual cognitive domains) and the risk of diabetes in older adults. This finding extends previous findings by suggesting the importance of cognitive function related to the risk of diabetes among older adults.

While previous studies have largely focused on how improvements in cognitive function can reduce the risk of developing dementia (Biessels and Whitmer, 2020; Srikanth et al., 2020), the current study expands this body of knowledge by investigating the reverse relationship—how cognitive decline increases the risk of diabetes. This novel approach contrasts with previous diabetes studies, which have primarily examined how diabetic symptoms such as glucose sensitivity and glycemic control affect cognitive decline and the risk of dementia (Campbell et al., 2012; Fang et al., 2019; Gregg et al., 2018; Gregg et al., 2014; Gordon-Dseagu et al., 2014; Seshasai et al., 2011). Even though this reverse relationship has not been conventional, it offers new insights into how cognitive decline can increase the risk of diabetes, challenging traditional perspectives in diabetes research. The findings of this study provide ample evidence for the importance of participating in programs and activities designed to enhance cognitive functioning in older adults. Additionally, it contributes to the broader discussion about the necessity of diverse therapeutic programs, which should include both traditional behavioral interventions such as physical activity and cognitive-stimulating activities to support cognitive health and reduce the risk of diabetes. For example, substantial evidence suggests that cognitive stimulation activities (e.g., crossword puzzles, reading, writing, and Sudoku) and physical activities are important interventions that can increase cognitive function and help delay or reduce the risk of developing dementia (Carlson et al., 2012; Gidicsin et al., 2015; Rajan et al., 2015; Rajan et al., 2019; Reed et al., 2011). Healthcare providers should focus their efforts on designing and implementing programs and activities that can improve cognitive functioning in older adults.

The inclusion of cognitive screenings in regular assessments enhances proactive health management for older adults at risk of cognitive issues related to diabetes (Athilingam et al., 2015; LeRoith et al., 2019). This approach encompasses assessments that consider both cognitive and metabolic parameters in routine care protocols using technology-assisted care systems such as multidomain apps. Multidomain approaches offer enhancing clinical assessments and rehabilitation by delivering detailed, objective, and personalized patient insights, and while challenges remain for widespread clinical adoption, they are crucial for advancing neurophysiological research and standard practice (Scano et al., 2023). Comprehensive monitoring and evaluation enhance the early detection of diabetes-related cognitive decline, facilitating prompt intervention and management strategies (Kim and Fritschi, 2021; Ochieng and Bonney, 2018). Healthcare professionals can gain a nuanced understanding of the health profile of an individual, which will facilitate the formulation of tailored care plans that holistically address both metabolic problems and cognitive dimensions. Furthermore, it is important to advocate for healthcare policies that address the relationship between chronic health challenges such as diabetes and cognitive wellbeing in older adults (Juanamasta et al., 2021; Willette et al., 2013).

### Limitations

The current study is subject to inherent limitations that can be addressed in future research. First, this study did not differentiate between type 1 and type 2 diabetes in the analysis. The HRS dataset collected information about whether respondents had ever been diagnosed with diabetes without specifying the type. We acknowledge that this study relies on the diagnosis of diabetes based on a single questionnaire rather than using biodata such as blood tests, and the precise time frame for the onset of diabetes between 2012 and 2020 is unclear. Furthermore, type 1 and type 2 diabetes may impact cognitive functioning differently in older adults. Moreover, the type of diabetes could impact the design of multidomain clinical interventions, including physical and cognitively stimulating activities. Future studies should further analyze the relationship between the risk of diabetes and cognitive function in different types of diabetes in older adults. Second, we used three cognitive tests to assess cognitive function but neglected other cognitive function domains. Although previous studies have focused on the three cognitive domains examined in this study (Wessels et al., 2011; Ravona-Springer et al., 2010; Maggi et al., 2009; Debette et al., 2011), our limited scope of investigation hampered our ability to explain the relationship between the risk of diabetes and cognitive function in domains such as perceptual-motor control and social cognition.

Finally, this study did not account for all possible confounders that might have affected the relationship between cognitive function and the risk of diabetes. Preexisting comorbidities (e.g., hypertension and cardiac disease), behavioral factors (e.g., physical activity, sleep, and nutrition), and compliance with medication are significant external and internal confounders that can affect the estimation of diabetes risk. In future research, implementing machine learning analysis, such as neural network models, would be helpful not only for incorporating confounders such as comorbidities, behavioral factors, and medication adherence but also for understanding the confounding structure that affects the relationship between cognitive function and diabetes risk.

Despite these limitations, this study contributes to filling gaps in the literature by exploring: (a) the association between each cognitive function and the decline in diabetes risk and (b) the varying patterns of change in diabetes risk with increasing cognitive function. The findings of this study indicate that higher levels of each cognitive function examined are associated with a reduced risk of diabetes. In addition, our study suggests that participation in innovative programs and activities that stimulate and increase cognitive functions will benefit older adults who seek to manage the risk of diabetes.

## Data Availability

Publicly available datasets were analyzed in this study. This data can be found here: https://hrs.isr.umich.edu/.
